# Data on species list and the amount of macrophytes and mobile epi-benthic invertebrates in a subtropical seagrass-seaweed mixed bed in Ishigaki Island, Japan

**DOI:** 10.1016/j.dib.2018.07.031

**Published:** 2018-07-19

**Authors:** Kenta Nakamoto, Jun Hayakawa, Tomohiko Kawamura, Masafumi Kodama, Hideaki Yamada, Takashi Kitagawa, Yoshiro Watanabe

**Affiliations:** a International Coastal Research Center, Atmosphere and Ocean Research Institute, The University of Tokyo, 2-106-1, Akahama, Otsuchi, Iwate 028-1102, Japan; b Seikai National Fisheries Research Institute, Japan Fisheries Research and Education Agency, 1551-8, Taira-machi, Nagasaki, Nagasaki 851-2213, Japan; c Atmosphere and Ocean Research Institute, The University of Tokyo, 5-1-5, Kashiwanoha, Kashiwa, Chiba 277-8564, Japan

**Keywords:** Seagrass, Seaweed, Mobile epi-benthic invertebrate, Seagrass-seaweed mixed bed

## Abstract

In April 2014 and 2015, field samplings were conducted in a subtropical seagrass-seaweed mixed bed in Ishigaki Island, southwest Japan in order to collect macrophytes and mobile epi-benthic invertebrates. This article describes macrophyte biomass of 16 species or groups and invertebrate abundance of 66 species or groups. This data is associated with “Phylogenetically diverse macrophyte community promotes species diversity of mobile epi-benthic invertebrates” (Nakamoto et al., 2018) [1]

**Specifications Table**

**Table:** 

Subject area	Biology
More specific subject area	Marine Ecology
Type of data	Tables
How data was acquired	Macrophytes were dried at 60 °C for 24–48 h; Mobile epi-benthic invertebrates were counted
Data format	Raw
Experimental factors	Haphazardly sampling of macrophytes and invertebrates using 50 cm×50 cm quadrats
Experimental features	Species list and the amount of macrophytes and mobile epi-benthic invertebrates were described for each quadrat
Data source location	Ishigaki Island, Okinawa, Japan (24°23׳ 23" N; 124°08׳ 05" E)
Data accessibility	All data are available in this article
Related Research Article	[1] K. Nakamoto, J. Hayakawa, T. Kawamura, M. Kodama, H. Yamada, T. Kitagawa, Y. Watanabe, Phylogenetically diverse macrophyte community promotes species diversity of mobile epi-benthic invertebrates, Estuar. Coast. Shelf Sci., 207 (2018) 56–62

**Value of the data**

• This data provides the species list of macrophytes and mobile epi-benthic invertebrates in a seagrass-seaweed mixed bed in Nagura Bay, Ishigaki Island, Japan.
• This data also includes the biomass of macrophytes and the abundance of mobile epi-benthic invertebrates.
• This data is useful for comparing flora and fauna with those in other places.

## 1 Data

The species list and the biomass of macrophytes collected in a subtropical seagrass-seaweed mixed bed in Nagura Bay, Japan in April 2014 and 2015 are given in Table S1. Table S2 shows the species list and the abundance of mobile epi-benthic invertebrates. The quadrate number in Table S1 corresponds to that of Table S2.

## 2 Experimental design, materials and methods

### 2.1 Study site

The survey was conducted in Nagura Bay, Ishigaki Island, Okinawa, Japan (24°23׳ 23" N; 124°08׳ 05" E). The depth of the study site is about 0.2 m at the spring ebb tide. Sandy bottoms broadly occur in the study site and seagrasses are found all year round. Seaweeds grow from winter to spring, and those biomass reaches a maximum in April. Seaweeds mainly attach to gravel and detrital coral as well as shells and those biomass appeared to be influenced by the abundance of hard substrata, which were patchily located on the sandy bottom, so that the biomass of seaweed also showed a patchy distribution.

Field samplings were conducted in April 2014 and 2015. Twelve quadrats (50 cm×50 cm) were haphazardly put on the bed at the points where the degree of macrophyte heterogeneities were different from each other, ranging from where almost only seagrasses grew to where seagrasses and seaweeds were densely mixed. No distinct environmental clines (e.g. water temperature, salinity, depth) were observed. In each quadrat, seagrasses and seaweeds were cut with scissors at sheaths or rhizomes just above the sand bottom or at rhizoids, respectively, and then collected in a net (0.8 mm mesh) with coexistent mobile epi-benthic invertebrates. All the samples collected were immediately put in a cool box filled with seawater and seawater ice at the sampling site, and were carried to the Research Center for Subtropical Fisheries, Seikai National Fisheries Research Institute and immediately frozen at −20 °C for preservation. Seagrasses and seaweeds were also classified to the lowest possible taxonomic level, and then dried at 60 °C for 24–48 h. Mobile epi-benthic invertebrates retained on a 1 mm-mesh sieve were identified to the lowest possible taxonomic level and the number of each invertebrate species was counted.
