# Supplementary material for: Data on species list and the amount of macrophytes and mobile epi-benthic invertebrates in a subtropical seagrass-seaweed mixed bed in Ishigaki Island, Japan
Source: Data Brief. 2018 Jul 19;19:2442–4. doi: 10.1016/j.dib.2018.07.031 (PMC6141488; doi:10.1016/j.dib.2018.07.031)
Supplement: Supplementary file 1 — Supplementary material [file mmc1.docx]

Conflict of interest

Regarding this data, there is no conflict of interest whatsoever with any individual, or group.
